# Deep learning enhances reliability of dynamic contrast-enhanced MRI in diffuse gliomas: bypassing post-processing and providing uncertainty maps

**DOI:** 10.1007/s00330-025-11588-z

**Published:** 2025-04-19

**Authors:** Young Wook Lyoo, Haneol Lee, Junhyeok Lee, Jung Hyun Park, Inpyeong Hwang, Jin Wook Chung, Seung Hong Choi, Jaejun Yoo, Kyu Sung Choi

**Affiliations:** 1 Department of Radiology, Seoul National University Hospital, Seoul, Republic of Korea; 2 Graduate School of Artificial Intelligence, Ulsan National Institute of Science and Technology (UNIST), Ulsan, Republic of Korea; 3 Interdisciplinary Programs in Cancer Biology Major, Seoul National University Graduate School, Seoul, Republic of Korea; 4 Department of Radiology, Seoul Metropolitan Government Seoul National University Boramae Medical Center, Seoul, Republic of Korea; 5 Department of Radiology, Seoul National University College of Medicine, Seoul, Republic of Korea

**Keywords:** Gliomas, Deep learning, Perfusion MRI, Pharmacokinetic modelling, Uncertainty maps

## Abstract

**Objectives:**

To propose and evaluate a novel deep learning model for directly estimating pharmacokinetic (PK) parameter maps and uncertainty estimation from DCE-MRI.

**Methods:**

In this single-center study, patients with adult-type diffuse gliomas who underwent preoperative DCE-MRI from Apr 2010 to Feb 2020 were retrospectively enrolled. A spatiotemporal probabilistic model was used to create synthetic PK maps. Structural Similarity Index Measure (SSIM) to ground truth (GT) maps were calculated. Reliability was evaluated using the intraclass correlation coefficient (ICC) for synthetic and GT PK maps. For clinical validation, Area Under the Receiver Operating Characteristic Curve (AUROC) was obtained for predicting WHO low vs high grade and IDH-wildtype vs mutant.

**Results:**

329 patients (mean age, 55 ± 15 years, 197 men) were eligible. Synthetic K^trans^, Vp, Ve maps showed high SSIM (0.961, 0.962, 0.890) compared to the GT maps. The ICC of PK maps was significantly higher in synthetic PK maps compared to the conventional approach: 1.00 vs 0.68 (*p* < 0.001) for K^trans^, 1.00 vs 0.59 (*p* < 0.001) for Vp, 1.00 vs 0.64 (*p* < 0.001) for Ve. PK values of enhancing tumor portion obtained from synthetic and GT maps were comparable in AUROC: (1) K^trans^, 0.857 vs 0.842 (*p* = 0.57); Vp, 0.864 vs 0.835 (*p* = 0.31); and Ve, 0.835 vs 0.830 (*p* = 0.88) for mutation prediction. (2) K^trans^, 0.934 vs 0.907 (*p* = 0.50); Vp, 0.927 vs 0.899 (*p* = 0.24); and Ve, 0.945 vs 0.910 (*p* = 0.24) for glioma grading.

**Conclusion:**

Synthetic PK maps generated from DCE-MRI using a spatiotemporal probabilistic deep-learning model showed improved reliability without compromising diagnostic performance in glioma grading.

**Key Points:**

***Question*** *Can a deep learning model enhance the reliability of dynamic contrast-enhanced MRI (DCE-MRI) for more consistent and clinically acceptable glioma imaging?*

***Findings*** *A spatiotemporal deep learning model outperformed the Tofts model in Ktrans reliability and preserved diagnostic performance for IDH mutation and glioma grade, bypassing arterial input function estimation*.

***Clinical relevance*** *Enhancing DCE-MRI reliability with deep learning improves imaging consistency, supports molecular tumor characterization through reproducible pharmacokinetic maps, and enables personalized treatment planning, which might lead to better clinical outcomes for patients with diffuse gliomas*.

**Graphical Abstract:**

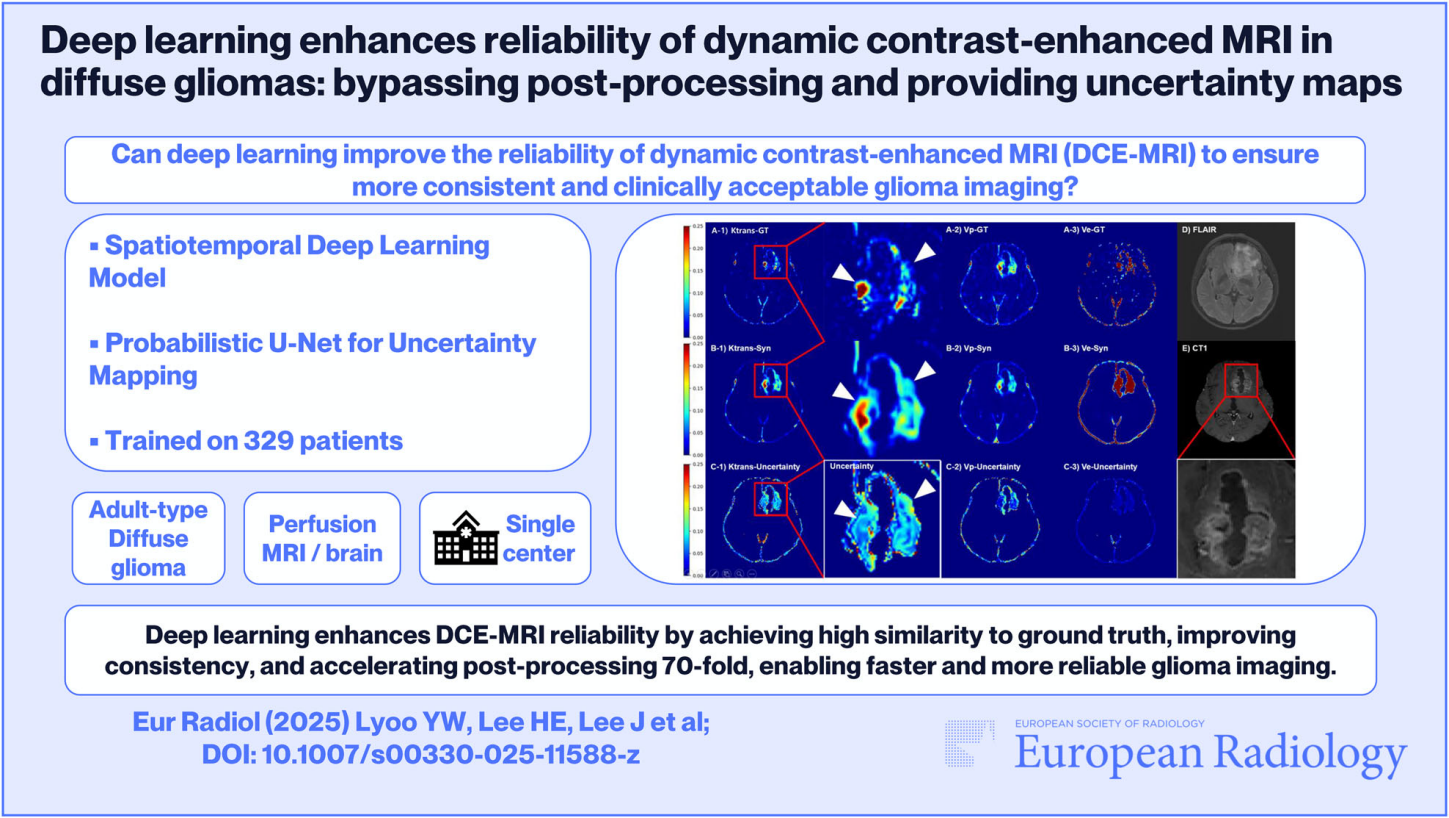

## Introduction

Dynamic contrast-enhanced magnetic resonance imaging (DCE-MRI) is a perfusion imaging sequence valuable in predicting the WHO grades of astrocytomas [1], and in distinguishing pseudoprogression [2] and radiation-induced effects [3] from true progression.

DCE-MRI uses dynamic variations in signal intensity that typify the tumor tissue and tracer kinetic models to generate pharmacokinetic (PK) parameters like the volume transfer constant (K^trans^), the fractional volume of the extravascular extracellular space (Ve), and the fractional volume of the vascular plasma space (Vp) [4]. These PK parameters reflect not only tumor angiogenesis (Vp) but also the permeability of microcirculation (K^trans^ and Ve). In neuroradiology, DCE-MRI has been less commonly used than dynamic susceptibility contrast MRI (DSC-MRI), an alternative perfusion imaging sequence. One critical factor in this lower acceptance is the low reliability of DCE-MRI [5–7]. The T1 signal intensity (SI) of DCE-MRI is inherently lower than the T2* SI of DSC-MRI, resulting in a lower signal-to-noise ratio. This leads to a noisy arterial input function (AIF). Additionally, the partial volume artifact contributes to the variability of the AIF [8]. The AIF is important in deriving the PK parameters, so the PK parameters also have low reliability [9].

A number of studies have attempted to address the low reliability of DCE-MRI using deep learning. A previous study used a conditional generative adversarial network to synthesize DSC-MRI-derived AIF from DCE-MRI, improving PK parameter reliability [10]. Another interesting approach leverages a convolutional neural network that directly estimates PK parameters from the raw DCE-MRI data, bypassing the need for estimation of the AIF [11]. This direct estimation approach also has the benefit of being less computationally extensive, as the conventional voxel-wise PK model fitting approach requires considering thousands of voxels for a single MR slice.

However, deep learning algorithms are often criticized for being black boxes and lacking interpretable explanations. Recent studies regarding chest radiographs focus on quantification of uncertainty. This drawback may have large consequences in decision-making applications, especially in the medical field. Uncertainty quantification methods, like the probabilistic U-Net, can be used to quantify and reduce the impact of uncertainties [12].

To overcome the limitations identified in DCE-MRI, we introduce and clinically validate a spatiotemporal approach that integrates a temporal convolutional network with a probabilistic U-Net. This hybrid model estimates PK parameters directly from DCE-MRI data without the need for AIF selection or PK model fitting and includes uncertainty estimation to enhance decision-making reliability.

## Materials and methods

### Patients

In this retrospective study, patients were consecutively enrolled from a single center, tertiary hospital. The Institutional Review Board (IRB) of Seoul National University Hospital approved this study (No. 2212-077-1385), and written informed consent was waived. From April 2010 to Dec 2020, patients over 18 years of age who underwent a treatment-naïve MRI using a 3-T glioma protocol consisting of DCE-MRI, T1-weighted imaging (T1WI), T2-weighted imaging (T2WI), and T2 fluid-attenuated inversion recovery (FLAIR) imaging were considered for inclusion.

**Fig. 1:**
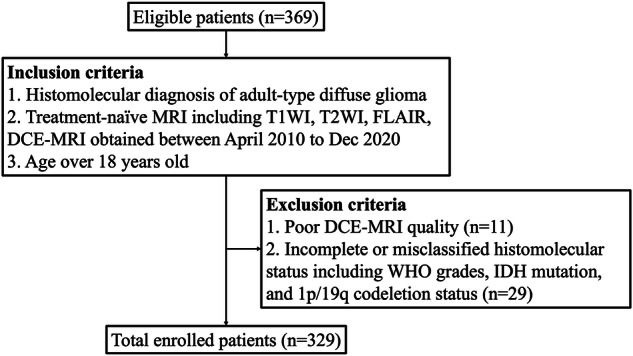
Flowchart of patient inclusion and exclusion criteria. T1WI, T1-weighted imaging; T2WI, T2-weighted imaging; DCE, dynamic contrast-enhanced; FLAIR, fluid-attenuated inversion recovery; IDH, isocitrate dehydrogenase; WHO, World Health Organization

Patients with histomolecular diagnoses of adult-type diffuse gliomas based on the 2021 WHO classification of tumors of the central nervous system were included: (1) histological glioblastoma, IDH-wildtype, histopathologic grade 4; (2) molecular glioblastoma, IDH-wildtype, histopathologic grade 2-3 with molecular alterations of either telomerase reverse transcriptase (TERT) promoter mutation, epidermal growth factor receptor (EGFR) amplification, or 7p+/10q- chromosomal copy number changes; (3) astrocytoma, IDH-mutant; and (4) oligodendroglioma, IDH-mutant, 1p/19q-codeleted [13], resulting in 369 patients. Patients with poor MRI quality (*n* = 11); incomplete or misclassified histomolecular status including WHO grades, IDH mutation, and 1p/19q codeletion status (*n* = 29) were excluded. A total of 329 patients met the inclusion criteria (Fig. 1). The dataset was temporally split so that the test set (102 patients) consisted of scans taken after March 2016. The remaining 219 patients were randomly split into the training set (165 patients) and the validation set (62 patients).

### Data collection and processing

The data supporting the findings of this study are included within the paper and its supplementary materials. Raw data can be obtained from the corresponding author upon reasonable request. The experimental code and 3D model are available on GitHub at: https://github.com/kyuchoi/dce2ktrans.

Motion correction and noise modulation were done on the raw DCE-MRI data. Ground truth (GT) PK maps were obtained using nordicICE 4.2.0 (NordicNeuroLab), a dedicated and widely accepted commercial software package. The DCE module of nordicICE was used to obtain the AIF. Two neuroradiologists (Radiologist 1 and Radiologist 2, with 20 and 8 years of experience, respectively) independently selected the AIF for each subject. The AIF was semi-automatically detected at the level of the horizontal segment of the middle cerebral artery to minimize subjectivity. K^trans^, Vp, Ve, and other physiological maps were then obtained by fitting to the extended Tofts model [4].

A glioma segmentation neural network algorithm (HD-GLIO) segmented T1WI-space registered T1WI, T2WI, FLAIR images to obtain tumor segmentation maps for statistical analysis [14, 15]. Details of data processing are available in Supplementary Materials.

### Deep learning model

**Fig. 2:**
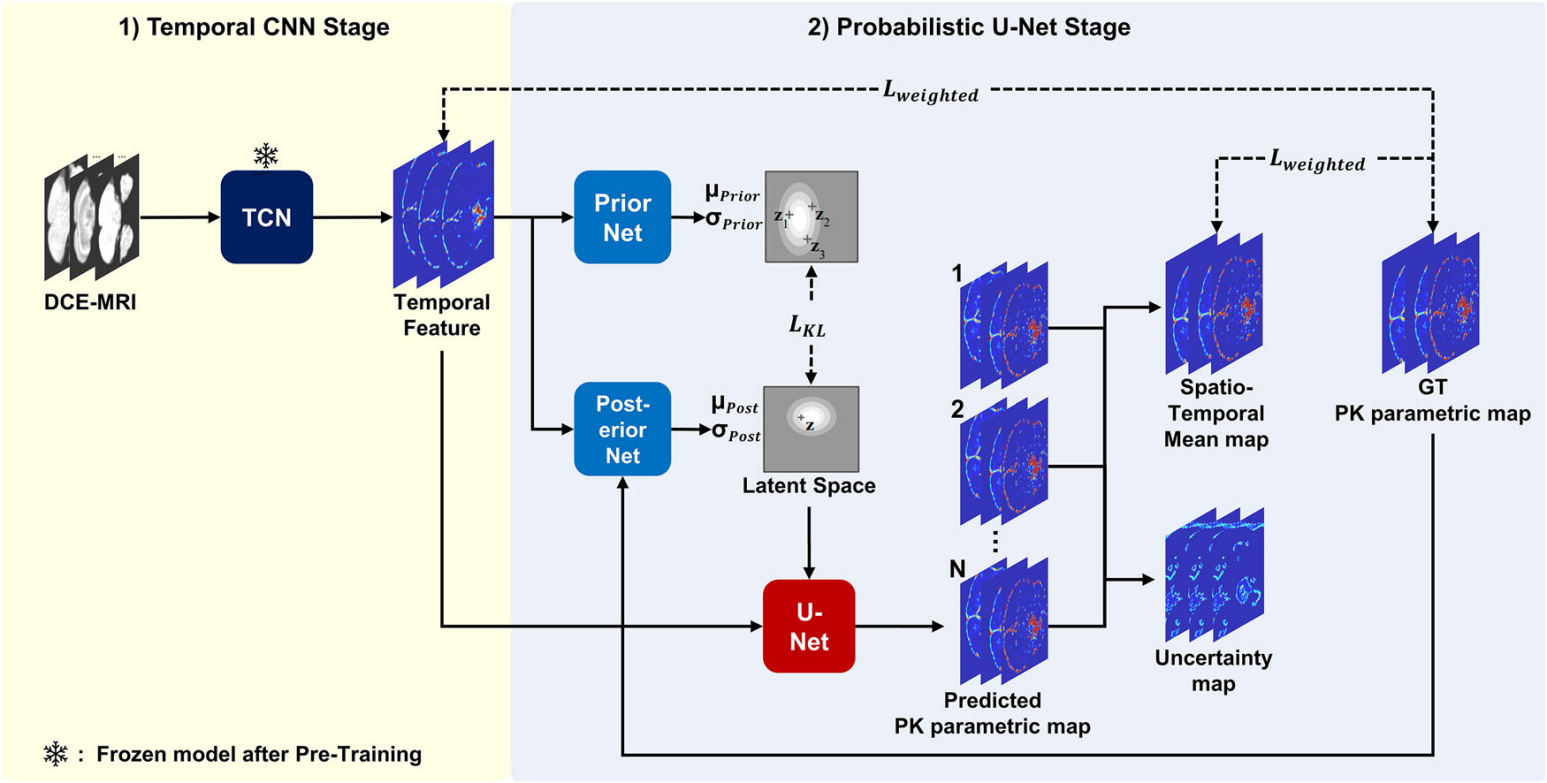
Network architecture overview. The neural network uses a two-stage architecture combining a temporal convolutional network and a probabilistic U-Net. A temporal convolutional network is applied to each voxel’s time curve, resulting in three parameter maps incorporating temporal information. These parameter maps are the input for a probabilistic U-Net, which incorporate spatial data in estimating the pharmacokinetic maps for K^trans^, Vp, Ve. The probabilistic U-Net also provides uncertainty maps that allow visualization of reliability at a pixelwise level. TCN, temporal convolutional network; GT, ground truth pharmacokinetic maps; K^trans^, volume transfer constant; Vp, fractional volume of the vascular plasma space; Ve, the fractional volume of the extravascular extracellular space

Accurately mapping DCE images to PK maps requires careful consideration of the underlying physics models. Given that the underlying physics model is primarily based on the analysis of voxel-wise time curves, we devised a spatiotemporal network architecture that incorporates voxel-wise analysis of temporal time curves as well as spatial data to improve the estimation of the PK maps. We use a serial 2-stage combination of a temporal convolutional network (TCN) [16] and the probabilistic U-Net [17] for the architecture (Fig. 2).

In the first stage, a TCN is used to condense the temporal information in the 60 time-point DCE maps into three temporal feature maps, one for each PK parameter. The TCN facilitates the extraction of critical features in the voxel-wise time curves. For the second stage, the temporal feature maps are used as inputs for a probabilistic U-Net. The probabilistic U-net enables integration of spatial information, while also providing uncertainty maps. Details on the deep learning model are available in Supplementary Materials.

### Experiments for validation

For quantitative validation of the model, Structural Similarity Index Measure (SSIM), and normalized root-mean-square-error (NRMSE) between the synthetic (predicted) and GT PK maps were calculated, for both tumor areas and total brain [18]. To compare the quantitative performance of temporal models and spatiotemporal models, an ablation study was done by comparing the full two-stage spatiotemporal model to a model with only the first-stage temporal 1D network. The SSIM, NRMSE values between the synthetic and GT PK maps were calculated (Table 1).

**Table 1:** Quantitative performance of deep learning models

Parameter	Metric	TCN	TCN + P-UNet	*p*-value
K^trans^	SSIM (Total)	0.948	0.961	< 0.001
K^trans^	NRMSE (Total)	3.505	2.657	< 0.001
K^trans^	NRMSE (Tumor)	16.27	14.82	0.04
Vp	SSIM (Total)	0.941	0.962	< 0.001
Vp	NRMSE (Total)	3.152	2.422	< 0.001
Vp	NRMSE (Tumor)	9.06	9.061	0.92
Ve	SSIM (Total)	0.816	0.890	< 0.001
Ve	NRMSE (Total)	8.547	6.053	< 0.001
Ve	NRMSE (Tumor)	43.64	37.06	0.02

Metrics are provided for (1) Temporal convolutional network (TCN) only, (2) Probabilistic U-Net (P-UNet) combined. Metrics were calculated between synthetic K^trans^, Vp, Ve maps and the ground truth maps. Synthetic maps showed high quantitative values of SSIM. The full two-stage spatiotemporal model using the TCN and the Probabilistic U-Net showed significant improvement in performance compared to a model with only the first-stage temporal 1D network

For SSIM, higher values indicate better performance. For NRMSE, lower values indicate better performance

*SSIM* structural similarity index measure, *NRMSE* normalized root mean squared error, *K*^*trans*^ volume transfer constant, *Vp* fractional volume of the vascular plasma space, *Ve* fractional volume of the extravascular extracellular space

Reliability of the PK maps was evaluated by comparing the intraclass correlation coefficient (ICC) for (1) conventional PK map generation using commercial software, (2) synthetic PK maps from the neural network model. In detail, the coefficient was calculated between two K^trans^, Vp, Ve measurements averaged for enhancing tumor ROIs. For conventional PK map generation, the ICC between PK maps was calculated for two independent selections of the AIF. For synthetic PK maps, the ICC between PK maps was calculated from the variability between 4 randomized prediction samples of the probabilistic U-Net.

For clinical validation, Area under the Receiver Operating Characteristic Curve (AUROC) values were calculated for predicting WHO grade (low-grade vs high-grade) and IDH mutation status using the PK maps. The conventional definition—grade 2 for low-grade, grades 3 and 4 for high-grade—was applied. Mean values of PK maps (K^trans^, Vp, Ve) were calculated within the enhancing tumor ROIs for each patient and used for prediction.

Post-processing time per patient was compared between the nordicICE commercial software and the two-stage spatiotemporal deep learning model. Statistics were averaged for all 102 patients from the test set. AIF selection was done by a single neuroradiologist. Inference for the deep learning model was done using a single NVIDIA RTX 3090 GPU. For the probabilistic U-Net, 4 random seeds were used. Total post-processing time was defined as time from loading the DCE volume to saving the PK maps. For nordicICE, this also includes the AIF selection time by the neuroradiologist. Inference time was defined as time directly spent calculating the PK maps, not including loading, displaying, or saving time.

### Statistical analysis

Continuous variables are summarized using means and standard deviations if normally distributed or as medians and interquartile ranges if nonnormally distributed. Quantitative variables were compared using Student’s *t*-test or the Mann–Whitney U test and categorical variables were compared using the χ^2^ or Fisher’s exact test. Bland-Altman plots were used to illustrate the agreement between synthetic and ground truth values for all PK parameters. *p*-values less than 0.05 were considered to be statistically significant, and all analyses were performed using R version 4.2.3. AUROC analysis was done with the pROC package [19].

## Results

### Patient characteristics

A total of 329 patients were included: 197 (60%) men, with a mean age of 55 ± 15 years. Among them, IDH-wildtype group was older than IDH-mutant group: mean age, 58 ± 15 vs 45 ± 13 (*p* < 0.0001). Pathology was comprised of glioblastoma, IDH-wildtype, histological grade 4 (*n* = 214, 65%); oligodendroglioma, IDH-mutant, and 1p/19q-codeleted (*n* = 32, 10%); astrocytoma, IDH-mutant (*n* = 50, 15%); and IDH-wildtype, histopathological grade 2 or 3 with molecular alterations either of TERT promoter mutation, EGFR amplification, or 7p+/10q- were considered to be molecular GBM (i.e., Glioblastoma, IDH-wildtype, WHO grade 4) (*n* = 33, 10%) according to the 2021 WHO Classification of Tumors of the Central Nervous System. Patients in the test set and the training/validation sets showed no statistically significant differences in age (*p* = 0.10), sex (*p* = 0.32), or tumor WHO grade (*p* = 0.30). However, there were significant differences in tumor IDH mutation status (*p* = 0.04) and tumor pathology (*p* < 0.001). Details on the characteristics of the test set and the train/valid sets are given in Table 2.

**Table 2:** Patient demographics and pathologic, genetic information in the training/validation set and the test set

Variables	Training/validation set	Test set	*p*-value
Number of patients	227	102	
Sex			0.322
Male, *n* (%)	140 (61.7)	57 (55.9)	
Female, *n* (%)	87 (38.3)	45 (44.1)	
Age (years), mean ± SD	54.3 ± 15.9	57.3 ± 14.2	0.103
WHO grade			0.297
WHO grade 2, *n* (%)	16 (7.0)	10 (9.8)	
WHO grade 3, *n* (%)	51 (22.5)	16 (15.7)	
WHO grade 4, *n* (%)	160 (70.5)	76 (74.5)	
Pathology			< 0.001
Glioblastoma, IDH-wildtype, *n* (%)	178 (78.5)	69 (67.7)	
Astrocytoma, IDH-mutant, *n* (%)	40 (17.6)	10 (9.8)	
Oligodendroglioma, IDH-mutant, 1p/19q codeleted, *n* (%)	9 (4.0)	23 (22.5)	
IDH mutation			0.037
Wildtype, *n* (%)	178 (78.4)	69 (67.6)	
Mutant, *n* (%)	49 (21.6)	33 (32.4)	

*p*-values are calculated using either the unequal variance *t*-test or the chi-square test

*IDH* isocitrate dehydrogenase, *WHO grade* Grades II, III, and IV represent World Health Organization grades

### Quantitative and qualitative validation of generation

In quantitative analysis of generation performance, synthetic PK maps (i.e., K^trans^, Vp, and Ve) showed high generative performance when compared to the GT PK maps: SSIM, 0.961, 0.962, and 0.890; NRMSE, 2.657, 2.422, and 6.053, respectively.

**Fig. 3:**
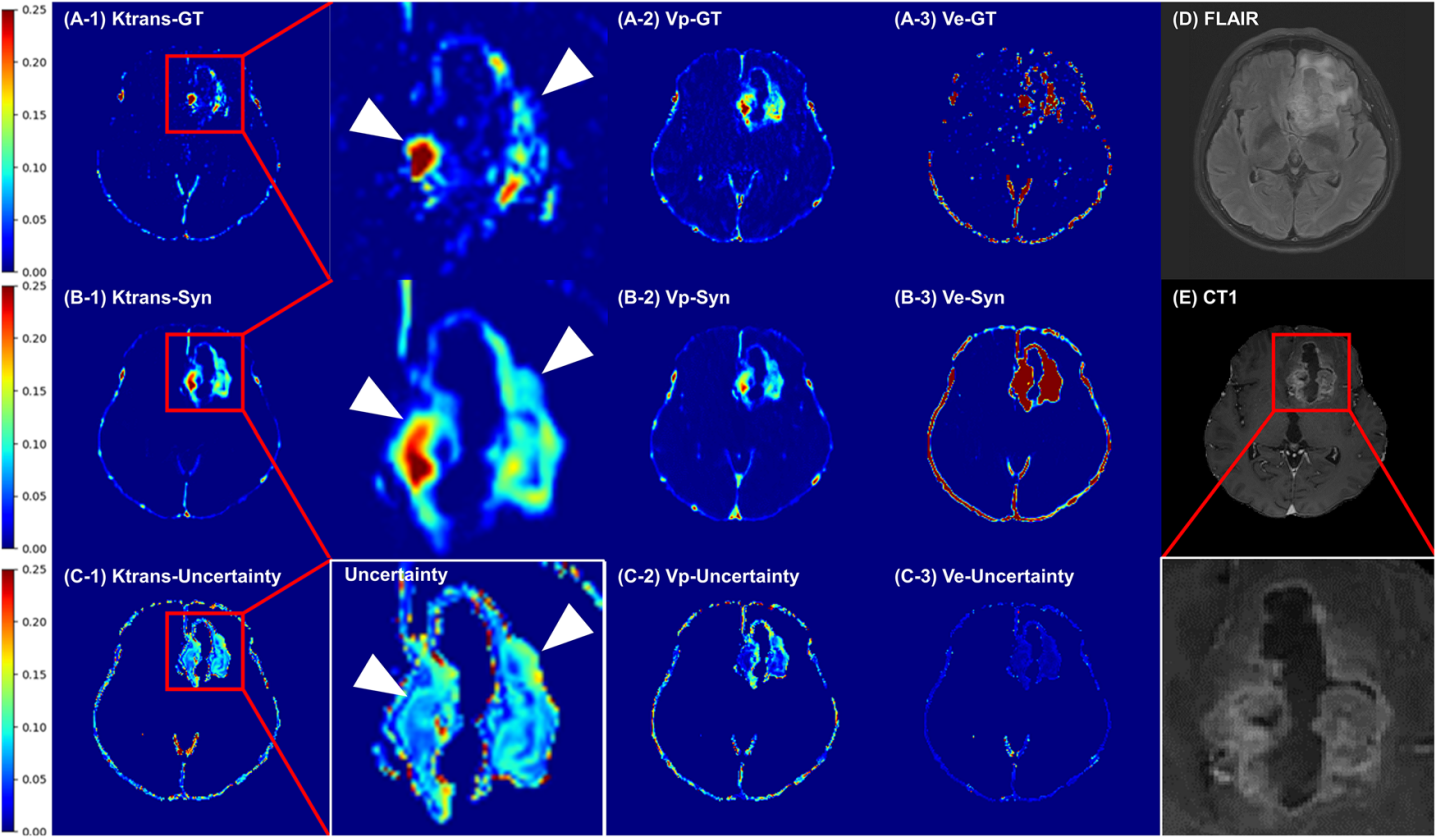
Comparison of ground truth and synthetic pharmacokinetic maps with matched uncertainty maps. A 61-year-old female patient with glioblastoma, IDH-wt, WHO grade 4 in the left frontal lobe. When magnified, the areas displaying gradual enhancement of the tumor periphery tend to exhibit high-uncertainty values: (**A**) Ground truth, (**B**) synthetic, and (**C**) uncertainty maps of K^trans^, Vp, and Ve maps, (**D**) T2 FLAIR image, (**E**) contrast-enhanced T1-weighted image. IDH, isocitrate dehydrogenase; FLAIR, fluid-attenuated inversion recovery; CT1, contrast-enhanced T1-weighted image; WHO grade, Grades II, III, and IV represent World Health Organization grades

**Fig. 4:**
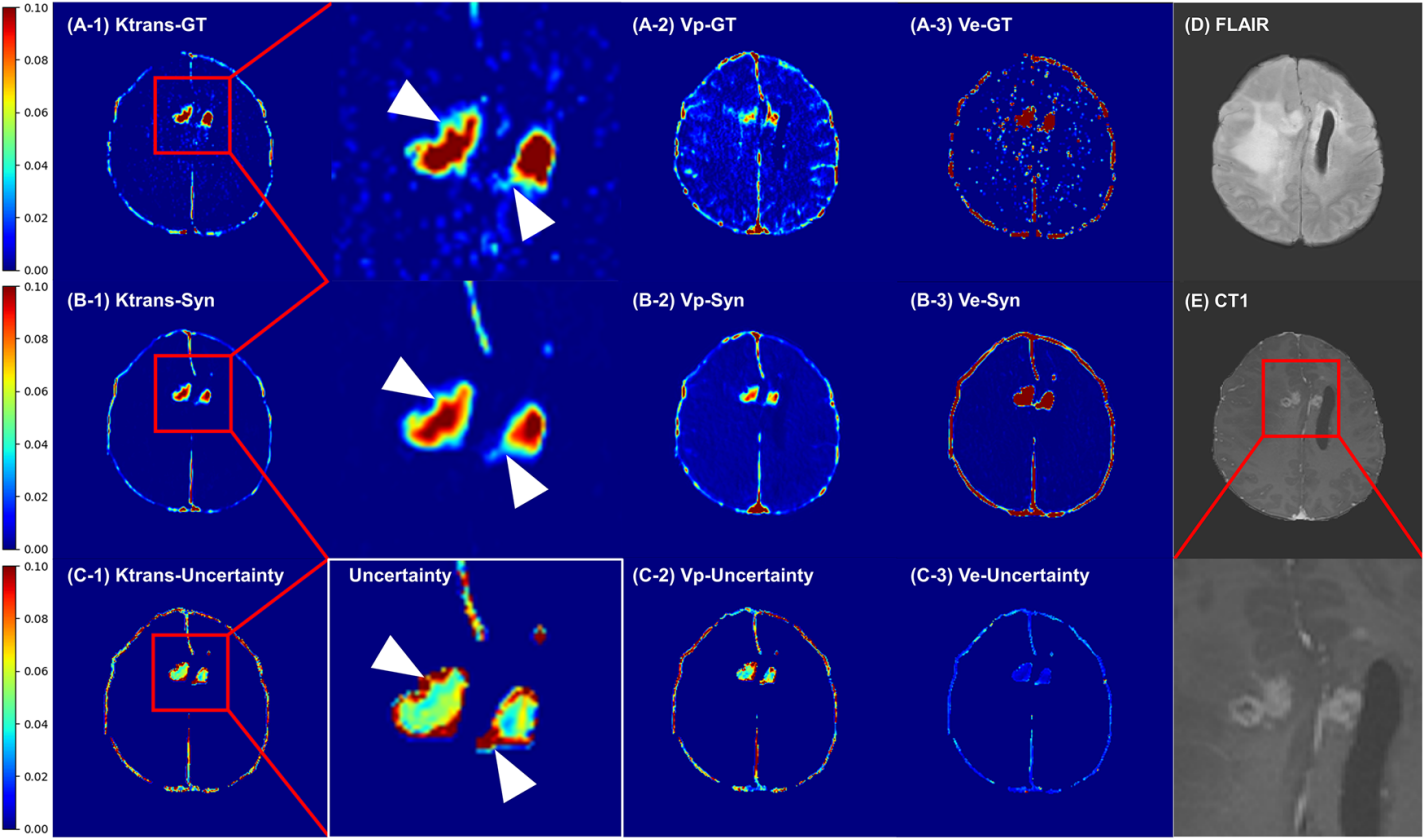
Comparison of ground truth and synthetic pharmacokinetic maps with matched uncertainty maps. A 44-year-old male patient with oligodendroglioma, IDH-mutant, WHO grade 3 in the bilateral frontal lobes. When magnified, the areas displaying gradual enhancement of the tumor periphery tend to exhibit high-uncertainty values: (**A**) Ground truth, (**B**) synthetic, and (**C**) uncertainty maps of K^trans^, Vp, and Ve maps, (**D**) T2 FLAIR image, (**E**) contrast-enhanced T1-weighted image. IDH, isocitrate dehydrogenase; FLAIR, fluid-attenuated inversion recovery; CT1, contrast-enhanced T1-weighted image; WHO grade, Grades II, III, and IV represent World Health Organization grades

Two representative cases demonstrate the qualitative effectiveness of the generated PK maps. Simultaneous examination of the uncertainty map indicates that the most unreliable areas are predominantly in the peripheral regions of the tumor, which display a gradual enhancement tracing from the outer to the inner tumor region (Figs. 3 and 4).

### Comparison of temporal vs spatiotemporal models

In the ablation study, the full two-stage spatiotemporal model showed higher performance compared to the one-stage 1D temporal model. For K^trans^, the SSIM was 0.961 vs 0.948 (*p* < 0.001). For Vp, the SSIM was 0.962 vs 0.941 (*p* < 0.001), and for Ve, the SSIM was 0.890 vs 0.816 (*p* < 0.001) (Table 1).

### Comparison of the reliability of PK maps

**Fig. 5:**
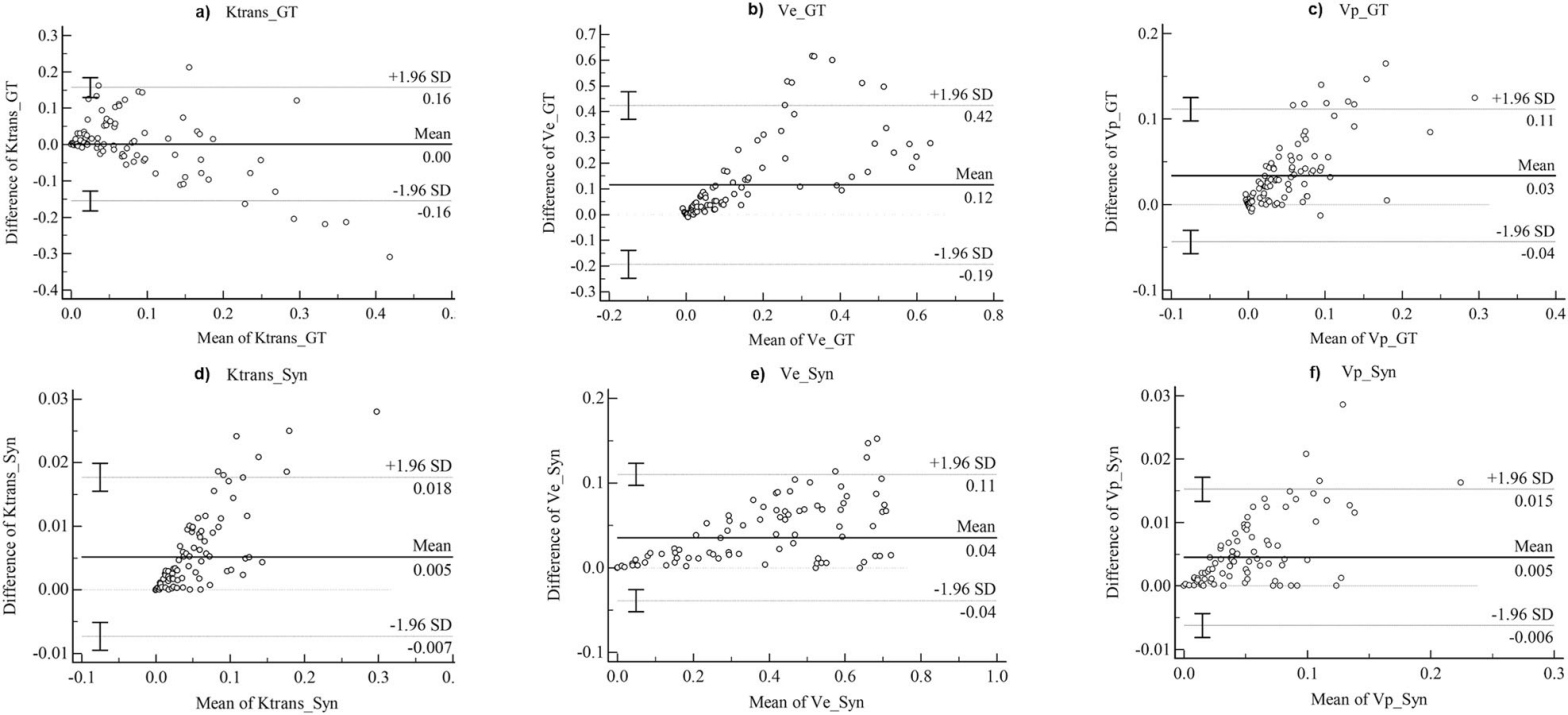
Bland-Altman plots for K^trans^, Ve, and Vp comparing the ground truth (upper) and synthetic (lower) pharmacokinetic parameters. Upper panels (**a**–**c**) display the ground truth plots—**a** K^trans^, **b** Ve, and **c** Vp—while lower panels (**d**–**f**) show the corresponding synthetic data plots—**d** K^trans^, **e** Ve, and **f** Vp. For all pharmacokinetic (PK) parameters (i.e., K^trans^, Ve, Vp), the synthetic PK parameters demonstrated improved agreement between two independent measurements compared to the ground truth (GT). Specifically, the mean differences were smaller in the synthetic PK parameters across all metrics. Additionally, the Bland-Altman plots indicated narrower limits of agreement—defined as the mean difference ± 1.96 times the standard deviation of the differences—for the synthetic PK parameters relative to the GT. Notably, the measurement ticks in the GT were approximately ten times larger than those in the synthetic PK parameters, further highlighting the enhanced consistency of the synthetic approach. Syn, synthetic; GT, ground truth pharmacokinetic maps; K^trans^, volume transfer constant; Vp, fractional volume of the vascular plasma space; Ve, fractional volume of the extravascular extracellular space

The reliability calculated using ICC was significantly higher for synthetic PK maps compared with the conventional approach. For K^trans^, the ICC was 1.00 (95% confidence interval (CI), 0.991–0.999) vs 0.68 (95% CI, 0.54–0.77) for synthetic vs GT PK maps (*p* < 0.001). For Vp, the ICC was 1.00 (95% CI, 0.992–0.998) vs 0.59 (95% CI, 0.43–0.71) (*p* < 0.001), and for Ve, the ICC was 1.00 (95% CI, 0.996–0.998) vs 0.64 (95% CI, 0.51–0.75) (*p* < 0.001). Bland-Altman plots demonstrated improved agreement for synthetic PK parameters compared to the GT, showing smaller mean differences and nearly tenfold narrower limits of agreement between two independent measurements (Fig. 5).

### Diagnostic performance for IDH mutation prediction task

**Fig. 6:**
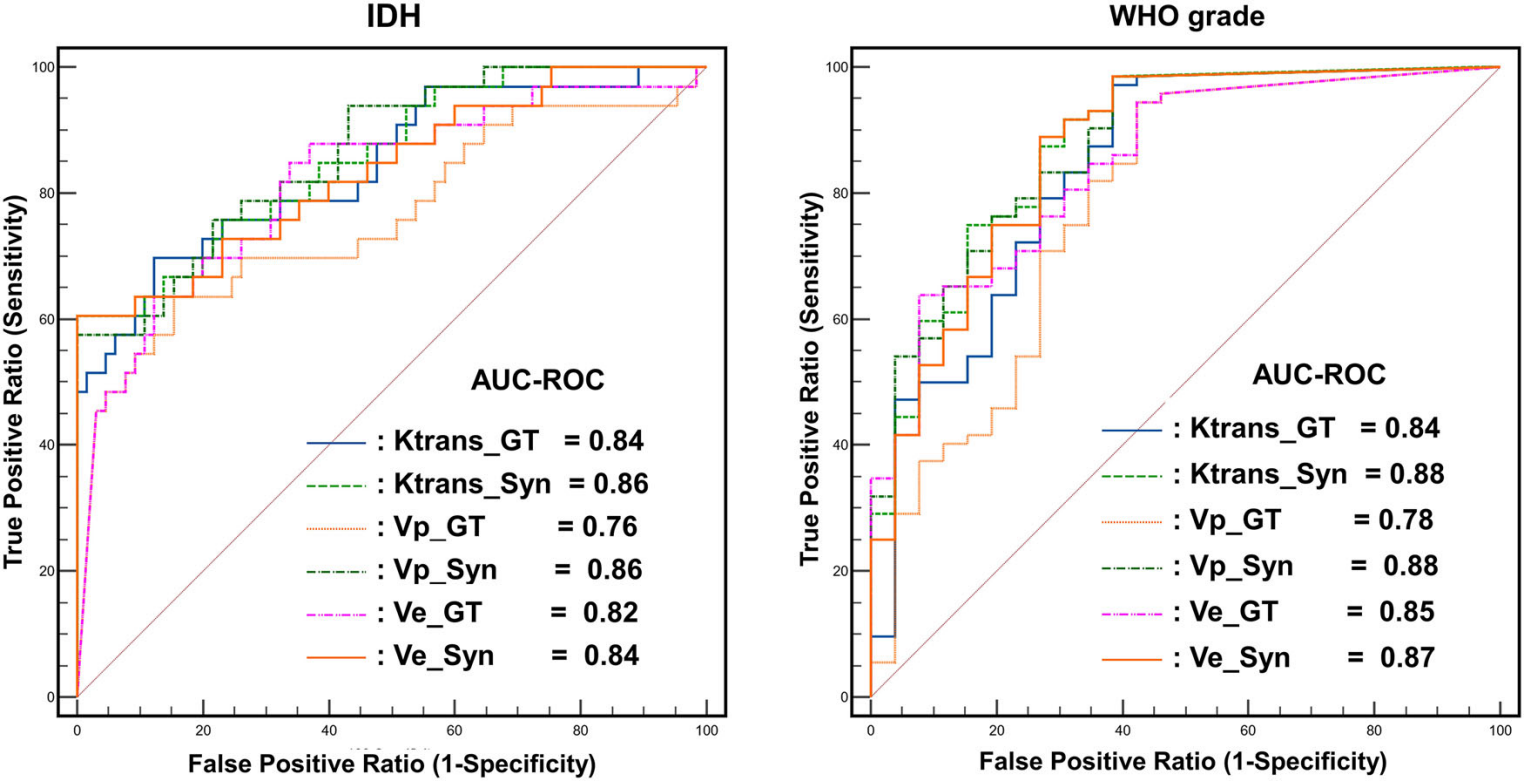
ROC curves of synthetic and ground truth PK parameters for the prediction of IDH mutation (right) and WHO grades (left). IDH, isocitrate dehydrogenase; WHO grade, Grades II, III, and IV represent World Health Organization grades

For the IDH mutation prediction task, PK values of enhancing tumor portion obtained from synthetic and GT PK maps were comparable. For K^trans^, the AUROC was 0.86 (95% CI, 0.77–0.92) vs 0.84 (95% CI, 0.75–0.91) for synthetic vs GT PK maps (*p* = 0.57). For Vp, the AUROC was 0.86 (95% CI, 0.78–0.93) vs 0.76 (95% CI, 0.67–0.84) (*p* = 0.009), and for Ve, the AUROC was 0.84 (95% CI, 0.75–0.90) vs 0.82 (95% CI, 0.73–0.89) (*p* = 0.70) (Fig. 6).

### Diagnostic performance for WHO grade prediction task

For the WHO grade prediction task (low-grade vs high-grade), PK values of enhancing tumor portion obtained from synthetic and GT PK maps were comparable. For K^trans^, the AUROC was 0.88 (95% CI, 0.80–0.94) vs 0.84 (95% CI, 0.75–0.91) for synthetic vs GT PK maps (*p* = 0.21). For Vp, the AUROC was 0.88 (95% CI, 0.80–0.94) vs 0.78 (95% CI, 0.68–0.86) (*p* = 0.02), and for Ve, the AUROC was 0.87 (95% CI, 0.78–0.93) vs 0.85 (95% CI, 0.75–0.91) (*p* = 0.64) (Fig. 6).

### Comparison of post-processing time

Post-processing time was markedly reduced using the two-stage spatiotemporal deep learning model when compared to nordicICE commercial software. Total post-processing time was 344.1 vs 4.7 s (*p* < 0.001), showing a 70-fold improvement. Model inference time was 62.1 vs 0.8 s (*p* < 0.001).

## Discussion

This retrospective study demonstrates how a spatiotemporal deep learning model can bypass PK model fitting post-processing, improve DCE parameter estimation reliability, and provide uncertainty estimates. Previous studies have used deep learning methods, including physics-informed neural networks (PINNs), to directly estimate PK parameters, overcoming computational time limitations of established PK models such as the Extended Tofts Model [11, 20, 21]. However, no studies have validated direct estimation using deep learning-based inference with a large-scale diffuse glioma dataset. Additionally, PINNs replicate the systematic errors of existing PK models since they use 1D voxel-wise modeling rather than true spatiotemporal multi-compartmental dynamic models [22]. Specifically, the Extended Tofts Model does not account for diffusion between neighboring voxels or intra- or inter-voxel exchange, and modeling with 1D voxels introduces constant systematic errors by relying on a global AIF assumption from the M1 segment of the middle cerebral artery, instead of a local AIF from the lesion tissue [23]. Furthermore, Buckley et al have already pointed out the issue of systematic errors [24]. Particularly in cases of low K^trans^, where the tumor is less visible and the signal-to-noise ratio is low, thus leading to high variance. This error significantly affects accuracy, posing a major barrier to reliable analysis and compromising key assumptions for quantitative imaging.

To address these issues, this study proposes using a probabilistic U-Net to provide uncertainty estimates along with PK parameters. A previous study by Bliesener et al proposed uncertainty estimation in DCE-MRI [25]. However, that study used only 1D curves, did not incorporate spatiotemporal dynamics, and utilized only simulated data with digital reference objects. This study is the first to apply such a model in both spatial and temporal dimensions to a large-scale patient group with diffuse glioma. Specifically, to learn the spatiotemporal dynamics of 4D DCE-MRI, the model structure is divided into two stages: one stage learns temporal features using a temporal convolutional network, and the other stage learns spatial features using a probabilistic U-Net. The ablation study with only the first-stage network shows that the spatiotemporal model achieves superior performance compared to the 1D temporal model in quantitative metrics such as SSIM and NRMSE.

The integration of deterministic and probabilistic networks can provide a robust and reliable system [26]. The temporal convolutional network offers advantages in extracting physical features; however it is a deterministic model that produces only a single specific result for a given input. This poses limitations, as retraining is the only option when false positives or negatives occur, restricting the model’s ability to capture real-world variability. In contrast, the Probabilistic U-Net utilizes Gaussian distributions in the latent space to produce multiple plausible synthesis results, which might resemble the variability seen in real-world PK parametric maps.

Synthetic DCE parameter maps exhibited high quantitative and qualitative similarity to the GT DCE parameter maps obtained using conventional methods. Moreover, these maps were generated significantly faster through the rapid inference capabilities of neural network models. Compared with synthetic K^trans^, Vp maps, synthetic Ve maps were less similar to the GT maps both quantitatively and qualitatively. This could possibly be due to the fact that GT Ve maps were inherently noisier than the GT K^trans^, Vp maps. We observed that the deep learning model seems to reduce noise in the parameter maps, and therefore noise differences were most pronounced for the Ve maps. Previous studies have revealed several systematic errors introduced by specific PK models: for example, Ve can be underestimated up to 300% in certain PK models [27], and for the Tofts model K^trans^ and Vp can be overestimated and underestimated, respectively [24].

For reliability, ICC values were significantly higher for the synthetic DCE parameter maps compared with the GT maps (1.00 vs 0.68 for K^trans^; 1.00 vs 0.59 for Vp; 1.00 vs 0.64 for Ve, all *p* < 0.001). We believe this is because the deep learning model is a fully automated approach that does not rely on an arbitrary choice of the AIF, which is known to be unreliable due to partial volume artifacts and motion artifacts. The comparison of LoA confirmed that the synthetic PK parameters demonstrated up to nearly tenfold improved agreement (Fig. 5). Although proportional bias was observed in the Bland-Altman plots for all PK parameters—an expected outcome given their proportion-based nature—this bias does not necessarily invalidate the bias estimates or the limits of agreement (LoA) [28, 29]. However, further research is warranted to explore alternative approaches, such as log transformation or different scaling, to mitigate proportional bias.

Interpreting uncertainty maps from a probabilistic model perspective, areas with high variance indicate relatively high uncertainty. These high-variance regions can be clinically interpreted in two perspectives: (1) They highlight areas where the model’s inference is ambiguous from the model’s standpoint, thus assisting clinicians in considering potential inaccuracies for more reliable interpretation during cancer diagnosis. For instance, in tumor areas with less enhancement, the uncertainty value increases as K^trans^ decreases (Figs. 3 and 4). Implementing the spatiotemporal uncertainty map, as suggested by the previous study’s future directions [25], we display parameter and uncertainty maps side-by-side, allowing the exclusion of high variance voxels to provide more reproducible imaging biomarkers by reducing error bounds; (2) Regardless of tumor type (oligodendroglioma, IDH mutant, or glioblastoma, IDH-wildtype), high-uncertainty regions often correspond to the peripheries of tumor areas exhibiting a gradual transition from non-enhancement to enhancement (Figs. 3 and 4). These regions would frequently mark potential resection margins prone to local recurrence/progression [30, 31], thereby assisting in identifying high-risk progression areas for patients [32]. From a deep learning perspective, generative models may “hallucinate,” leading to false-positive findings if the MR images are noisy. Estimating uncertainty, as our model demonstrates, is crucial for mitigating this issue by filtering out high-uncertainty voxels in the final interpretation.

Clinical validation was conducted to assess the diagnostic performance of synthetic and GT PK maps (K^trans^, Vp, Ve) in predicting IDH mutation status and WHO grade. This analysis generated 12 AUROC curves (Fig. 6). AUROC indicated good diagnostic performance (0.80–90) for 10 of the 12 cases. Lower AUROC values were observed while using GT Vp to predict IDH mutation status (AUROC = 0.76) and using GT Vp to predict WHO grade (AUROC = 0.78).

Pairwise comparisons of diagnostic performance were conducted between the synthetic and GT PK maps. Synthetic Vp maps demonstrated superior performance relative to the GT Vp maps for both the IDH mutation status prediction task (AUROC 0.86 vs 0.76, *p* = 0.009) and the WHO grade prediction task (AUROC 0.88 vs 0.78, *p* = 0.02). In contrast, no statistical difference in diagnostic performance was observed for K^trans^ and Ve for either task.

A possible explanation for the difference between synthetic Vp maps over GT Vp maps is that Vp tends to be systematically underestimated in the conventional PK models [24]. In our study, we also observed that Vp tended to show higher variance compared with K^trans^ and Ve in the uncertainty map (Fig. 3). This unreliability is possibly averaged out in the synthetic model, resulting in a substantial improvement in diagnostic capability.

Post-processing time was up to 70 times faster using the deep learning model compared to the conventional approach. This can be attributed to two factors. First, the deep learning model does not require human labeling of the AIF, which usually takes more than half of the total processing time. Second, the deep learning model is less computationally extensive, because the conventional approach has to fit thousands of voxels for a single MR slice simultaneously.

Statistical analyses revealed significant differences between the test set and the training/validation set regarding tumor IDH mutation status (*p* = 0.04) and tumor pathology (*p* < 0.001). These differences arise because we employed a temporal split instead of a random split. While a random split may yield higher model performance, a temporal split provides a more realistic assessment of model performance in real-world applications. This approach aligns with the objective of ensuring the model’s applicability and robustness in clinical settings.

Future studies should examine cases with low K^trans^ values, where tumors are less visible, particularly in about 7% of IDH-wildtype with “low-grade appearance” cases that appear less aggressive and have a favorable prognosis [33]. Resection of nonenhancing tumors, such as supramaximal resection, has shown potential to improve survival rates, though no specific guidelines exist for resection margins due to potential neurological deficits. DCE-MRI could facilitate studies on resection margins by guiding areas of angiogenesis in nonenhancing regions. However, the low enhancement can lead to poor tumor delineation in PK maps, reducing the reliability of parameters like K^trans^, Ve, and Vp. The use of uncertainty maps proposed in this study might mitigate systematic errors by allowing the exclusion of high-variance voxels, thus summarizing key biomarkers within their error bounds. Further studies are needed to test the model’s performance in broader clinical contexts, including postoperative and post-radiation therapy images, and for other pathologies. The model will require a large and diverse training dataset and minor adjustments to handle different DCE-MRI parameters.

There are several limitations to this study. First, external validation was not performed, as the study was conducted retrospectively at a single center, limiting the generalizability of the findings to other imaging protocols and populations. Second, the synthetic pharmacokinetic (PK) maps were validated against ground truth maps derived solely from the Tofts model, which has inherent limitations and may introduce bias. Lastly, while this study focused on the Tofts model, future work should incorporate other pharmacokinetic models, such as the Patlak model, into the network architecture to improve the generalizability and applicability of uncertainty learning in DCE-MRI.

In summary, the proposed spatiotemporal probabilistic deep-learning model for generating PK maps from DCE-MRI not only enhances reliability but also reduces post-processing time. It maintains or even improves diagnostic performance in predicting IDH mutations/WHO grades and provides voxel-wise guidance for interpreting PK maps through uncertainty maps. This effectively reduces the major obstacle of unreliability in quantitative imaging, which could be essential for treatment planning.

## Supplementary information

ELECTRONIC SUPPLEMENTARY MATERIAL

## Abbreviations

AIF: Arterial input function
DCE-MRI: Dynamic contrast-enhanced magnetic resonance imaging
GT: Ground truth
ICC: Intraclass correlation coefficient
IDH: Isocitrate dehydrogenase
K^trans^: Volume transfer constant
PK: Pharmacokinetic
SI: Signal intensity
Ve: Fractional volume of extravascular extracellular space
Vp: Fractional volume of vascular plasma space
