# Supplementary material for: Deep learning enhances reliability of dynamic contrast-enhanced MRI in diffuse gliomas: bypassing post-processing and providing uncertainty maps
Source: Eur Radiol. 2025 Apr 19;35(10):6229–39. doi: 10.1007/s00330-025-11588-z (PMC12417281; doi:10.1007/s00330-025-11588-z)
Supplement: Supplementary file 1 — ELECTRONIC SUPPLEMENTARY MATERIAL [file 330_2025_11588_MOESM1_ESM.pdf]

Deep Learning Enhances Reliability of Dynamic Contrast-Enhanced MRI in Diffuse Gliomas: Bypassing Post-processing and Providing Uncertainty Maps

ELECTRONIC SUPPLEMENTARY MATERIAL

Materials and Methods

MRI acquisition

MRI scans were performed using either one of two 3T MRI units (Magnetom Verio or Magnetom Skyra; Siemens Healthineers) with a 32-channel head coil.

Patients underwent a “glioma study” protocol that includes DCE-MRI, pre and post-contrast enhanced magnetization-prepared rapid acquisition gradient-echo (MP-RAGE) T1WI, axial T2WI, and axial T2- fluid attenuated inversion recovery (FLAIR) sequences.

DCE-MRI was acquired using a 3D T1-weighted spoiled gradient-echo sequence with the following scan parameters: repetition time msec/echo time msec, 2.8/1.0; flip angle, 10°; matrix, 192 × 192; field of view, 240 × 240 mm; section thickness, 3 mm; voxel size, 1.25 × 1.25 × 3 mm^3. 60 acquisitions were made with a temporal resolution of 4 seconds. DCE-MRI was performed using the aforementioned parameters after intravenous administration of gadobutrol (Gadovist; Bayer Schering Pharma) [at a dose of 0.1 mmol/kg]), followed by a 30-mL saline bolus at a rate of 4 mL/sec by using a power injector. (Spectris, MEDRAD). The total acquisition time was 5 minutes and 8 seconds.

For MP-RAGE, the following scan parameters were used: repetition time msec/echo time msec, 1370–1600/1.9–2.8; flip angle, 9°; matrix, 256 × 232; field of view, 250 × 250; section thickness, 1 mm; number of excitations, one. For axial T2-WI: repetition time msec/echo time msec, 5100/89 msec; flip angle, 150°; matrix, 640 × 348; field of view, 199 × 220; section thickness, 5 mm; number of excitations, three. For axial FLAIR: repetition time msec/echo time msec, 8000– 9000/90–97; inversion time, 2300–2500 msec; flip angle, 130–150°; matrix, 384 × 209–278; field of view, 199 × 220; section thickness, 5 mm; number of excitations, 1–2.

Data processing

All DICOM files were converted into NIfTI gzipped files. All images were motion corrected and N4 bias field corrected. The time-averaged DCE image was skull stripped. DCE images were resampled to resolution 256x256 in the xy-plane, resulting in a final resolution of 256x256x40x60.

Tumor segmentation was done using the following steps: First, T1WI, T2WI, FLAIR images were skull stripped using the BET package. Then, T1WI, T2WI, FLAIR images were centered, re-oriented to the standard neurological conventional (RAS+) orientation, and were resampled to isotropic 1mm voxels in xy-plane. T2WI and FLAIR images were registered to T1WI space using the nonlinear SyN algorithm from the ANTs package [13]. A glioma segmentation neural network algorithm (HD-GLIO) was used on the registered T1WI, T2WI, FLAIR images to obtain tumor segmentation maps [14]. Tumor segmentation maps were registered to DCE space using a rigid affine transform from the ANTs package, with the time-averaged resampled DCE image as the target.

Deep Learning Model

The network is trained in a two-stage process, rather than end-to-end. For the first stage, we train a temporal convolutional network to condense the temporal information in the 60 time-point DCE maps into three temporal feature maps, one for each PK parameter. The temporal convolutional network operates on each voxel independently, transforming a 1x60 vector into a 1x3 vector.

For the second stage, we use the temporal feature maps as the input for U-Net. The U-Net's capacity for learning spatial features using convolutions enables integration of spatial information to create reliable PK maps. The U-Net has also been widely used for tumor segmentation, so prediction of tumor presence is possible in a pixelwise fashion, potentially improving performance of PK map estimation. To bolster the reliability of the final PK maps, we specifically use the probabilistic U-Net architecture. The probabilistic U-net employs a conditional variational autoencoder approach capable of efficiently generating numerous plausible hypotheses. By integrating PriorNet and PosteriorNet, our network can derive an extensive array of randomized prediction samples. The prediction maps are iteratively generated multiple times (N=4), with the resulting mean of these iterations serving as the definitive PK map. The standard deviation values obtained from these iterations are used to calculate the uncertainty map. This strategy offers valuable insights into the diversity of predictions, ultimately enhancing the overall reliability of our predictions.

In detail, the 4D DCE images (256x256x40x60) were split along the z-axis into 40 3D images (256x256x60). Similarly, the PK map labels (256x256x40x3) were split along the z-axis into 40 3D images (256x256x3). The temporal convolutional network uses a kernel size of 7, dilated temporal convolutions with upscaling factor 2, and two intermediate channels of size 32. This takes as input 3D images (256x256x60), and operates along each pixel (256x256), resulting in a (256x256x3) temporal feature map. For the backbone of the

probabilistic U-Net, use 1 input channels, 3 output channels, and downsampling factor of 2 with a total depth of 7. This network takes as input (256x256x1) temporal feature map and output images of size (256x256x3), resulting in prediction maps for K^{trans}, V_p, V_e . The probabilistic U-Net is used to generate uncertainty maps as well the mean prediction maps for K^{trans}, V_p, V_e.

Model training, validation, and test procedures were conducted on dedicated workstation equipped with four NVIDIA RTX 3090 (NVIDIA) graphical processing units. For calculation of post-processing time, model inference was done using one NVIDIA RTX 3090 graphical processing unit. Code was implemented with PyTorch v1.7.0. The Adam optimizer with an initial learning rate of 0.0003 was used for training the model. A weighted-L1 loss that used the labels as linear weighting was used ($\mathrm{wL1}(y, x) = \frac{1}{N}\sum_{i=1}^{N} y_i|x_i - y_i|$) This emphasizes differences between the label and prediction in the case where the label value is large. Because large values of K^{trans}, V_p, V_e are often observed in tumor, we found that the weighted L1 loss was better than the conventional L1 loss in prediction of PK maps inside tumor segmentation maps.

The model training was performed on a single NVIDIA GeForce RTX 3090 GPU and consisted of two stages. The first stage, utilizing Temporal Convolution Networks, required 4 hours and 59 minutes to complete 58 epochs. The second stage, which employed the PUNET model, took 7 hours and 40 minutes to complete 63 epochs. Altogether, the training for both stages spanned approximately 12 hours and 39 minutes. Early stopping was applied to terminate training if the validation loss did not improve over 10 consecutive epochs.
